# Preparation, Structure and Characterization of Polymer/Cement Composites

**DOI:** 10.3390/polym15112495

**Published:** 2023-05-29

**Authors:** Bowen Guan

**Affiliations:** School of Materials Science and Engineering, Chang’an University, Xi’an 710061, China; bguan@chd.edu.cn

Polymer/cement composites have gained significant attention in civil engineering due to their improved properties compared to traditional cement-based materials. Due to the rapid advancement of polymer/cement composites and extensive research on the correlation between material structure and properties, the utilization of polymer/cement composites has increased significantly. As a result, research on this composite has become a critical area of focus both in China and worldwide. Polymer/cement composites have been used in various civil engineering applications.

Polymer/cement composites are used to improve the performance of pavement materials. For example, Xu et al. adopted a super absorbent polymer (SAP) as an internal curing agent to enhance the durability of pavement concrete [1]. Chen et al. added carboxyl butyl benzene latex and polyformaldehyde fibers in the concrete materials prepared for ultra-thin whitetopping (UTW) to improve the crack resistance and impact resistance [2]. As a type of organic-inorganic composite material, the performance of asphalt pavement material is closely related to its composition. The skid resistance of pavements can be improved using 50% steel slag content [3]. Li et al. reutilized two types of waste plastic (polypropylene (PP) and polyethylene (PE)) as asphalt modifiers to improve the performance of asphalt pavements [4]. Feng et al. found that nano-OvPOSS can be employed as a viable asphalt modifier to ensure a well-rounded performance of modified asphalt [5].

Polymer is a material that has revolutionized the way concrete is used in civil engineering. By adding polymers to concrete, the resulting material has improved performance in terms of strength, durability and resistance to water and chemical damage. Papán, D. et al. studied the material properties of FC500 foam concrete, the definition of standardized stress–strain diagrams of the FC500 material for compression and especially tension [6]. Chen et al. found that PVA improves the shrinkage of the UHCP [7]. Fly ash and Hexadecyltrimethoxysilane can improve compressive properties and water resistance of magnesium oxychloride cement [8].

Polymer-based soil stabilization is a popular technique in civil engineering that enhances the performance of soil. Polymers are long-chain molecules that can improve the physical and chemical properties of soil, such as its strength, stability and water retention capacity. Zhao et al. used Poly (Vinyl Alcohol) Solution and Silica Fume to enhance the strength of coarse-grained soil [9]. Sodium carboxymethyl cellulose (CMC) can improve the production capacity of saline–alkali soil [10]. Zhang et al. found that a 0.04% PAM dosage could improve soil hydrodynamic characteristics under brackish water infiltration, which is beneficial for the efficient utilization of brackish water [11].

In conclusion, polymer/cement composites have emerged as a promising material for various civil engineering applications due to their improved properties and performance compared to traditional cement-based materials. With ongoing research and development in this field, the use of polymer/cement composites is expected to increase in the future.
